# Epigenetic modulation of *AREL1* and increased *HLA* expression in brains of multiple system atrophy patients

**DOI:** 10.1186/s40478-020-00908-7

**Published:** 2020-03-09

**Authors:** Rasmus Rydbirk, Jonas Folke, Florence Busato, Elodie Roché, Alisha Shahzad Chauhan, Annemette Løkkegaard, Anne-Mette Hejl, Matthias Bode, Morten Blaabjerg, Mette Møller, Erik Hvid Danielsen, Tomasz Brudek, Bente Pakkenberg, Jorg Tost, Susana Aznar

**Affiliations:** 1 Research Laboratory for Stereology and Neuroscience, Bispebjerg-Frederiksberg Hospital, University Hospital of Copenhagen, Nielsine Nielsens Vej 6B, stair 11B, 2nd floor, DK-2400 Copenhagen, Denmark; 2 Laboratory for Epigenetics and Environment, Centre National de Recherche en Génomique Humaine, CEA-Institut de Biologie Francois Jacob, 2 rue Gaston Crémieux, FR-91000 Evry, France; 3 Copenhagen Centre for Translational Research, Bispebjerg-Frederiksberg Hospital, University Hospital of Copenhagen, Nielsine Nielsens Vej 4B, DK-2200 Copenhagen, Denmark; 4 Present address: Biotech Research and Innovation Centre, Faculty of Health, University of Copenhagen, DK-2200 Copenhagen, Denmark; 5 Department of Neurology, Bispebjerg-Frederiksberg Hospital, University Hospital of Copenhagen, Ebba Lunds Vej 44, DK-2400 Copenhagen, Denmark; 6 Institute of Clinical Medicine, Faculty of Health, University of Copenhagen, Blegdamsvej 3B, DK-2200 Copenhagen, Denmark; 7 Department of Neurology, Odense University Hospital, J.B. Winsløws Vej 4, DK-5000 Odense, Denmark; 8 Department of Clinical Research, University of Southern Denmark, DK-5000 Odense, Denmark; 9 Department of Neurology, Aarhus University Hospital, DK-8200 Aarhus, Denmark

**Keywords:** Multiple system atrophy, EWAS, Brain, Immune system, Hydroxymethylation, Neuroinflammation

## Abstract

Multiple system atrophy (MSA) is a rare disease with a fatal outcome. To date, little is known about the molecular processes underlying disease development. Its clinical overlap with related neurodegenerative movement disorders underlines the importance for expanding the knowledge of pathological brain processes in MSA patients to improve distinction from similar diseases. In the current study, we investigated DNA methylation changes in brain samples from 41 MSA patients and 37 healthy controls. We focused on the prefrontal cortex, a moderately affected area in MSA. Using Illumina MethylationEPIC arrays, we investigated 5-methylcytosine (5mC) as well as 5-hydroxymethylcytosine (5hmC) changes throughout the genome. We identified five significantly different 5mC probes (adj. *P* < 0.05), of which one probe mapping to the *AREL1* gene involved in antigen presentation was decreased in MSA patients. This decrease correlated with increased 5hmC levels. Further, we identified functional DNA methylation modules involved in inflammatory processes. As expected, the decreased 5mC levels on *AREL1* was concordant with increased gene expression levels of both *AREL1* as well as MHC Class I *HLA* genes in MSA brains. We also investigated whether these changes in antigen-related processes in the brain associated with changes in peripheral mononuclear cells. Using flow cytometry on an independent cohort of MSA patients, we identified a decrease in circulating non-classical CD14^+^CD16^++^ blood monocytes, whereas T and NK cell populations were unchanged. Taken together, our results support the view of an active neuroimmune response in brains of MSA patients.

## Introduction

Multiple System Atrophy (MSA) is a detrimental disease with no treatment possibilities. It is a neurodegenerative disease of the alpha-synucleinopathies where alpha-synuclein accumulates in both neurons and oligodendrocytes of the brain as neuronal or glial cytoplasmic inclusions, respectively [1, 2]. The mean onset is 55–60 years of age with an estimated survival time of 6 years [3]. The exact aetiology of MSA is unknown. The involvement of genomic factors in MSA development has been investigated [4], but so far the results have been inconclusive. This may correlate well with an estimated heritability of MSA below 7% [5]. Other mechanisms such as epigenetic changes may better explain development of MSA as they are proposed to causally reflect genetic-environmental interactions [6]. Epigenetic changes to the DNA have long been suspected to play a role in neurodegenerative diseases, including MSA [7, 8], but also Alzheimer’s disease (AD) [9, 10], Parkinson’s disease (PD) [11, 12], and Progressive Supranuclear Palsy (PSP) [13]. Specifically, 5-methylcytosine (5mC) is recognized as an important regulator of gene expression [14], but is not the only DNA-based epigenetic modification. 5-Hydroxymethylcytosine (5hmC), another equally important epigenetic regulator, is widely distributed in the brain [15] and is implicated in fetal brain development [16] as well as in different brain disorders [17, 18]. Disease-specific differences in global 5mC and 5hmC levels have been reported in selected areas of the brain in MSA patients by immunodetection [19]. However, the region-specific differences in global 5mC methylation levels could not be replicated in a recent array-based study as analysis of 5hmC was not performed [8]. Therefore, more information on the epigenetic landscape in MSA is required in order to infer on fundamental biological functions involved.

In the current study, we performed an epigenome-wide association study (EWAS) on prefrontal cortex brain tissue from 41 MSA patients and 37 normal, healthy controls (CTRLs). We utilized the Infinium MethylationEPIC array which allows for analyses of more than 850,000 methylation sites (CpGs) analysing both 5mC and 5hmC levels at single-nucleotide resolution. Our study thus represents the first detailed assessment of DNA methylation and hydroxymethylation in MSA brains. The aim of the study was to identify gene-specific epigenetic changes as well as the affected biological functions. From previous studies, we know that the prefrontal cortex is affected in MSA [20]. We validated the results from the BeadChip using NGS-based amplicon sequencing and performed RT-qPCR to confirm gene expression alterations of immune related components. Finally, in a novel cohort of 24 MSA patients and 46 CTRLs we validated the involvement of these immune-related changes during the disease course by investigating alterations in the composition of peripheral blood immune cells.

## Materials and methods

### Patient material

In the current study, 78 samples from the dorsomedial prefrontal cortex from frozen human brains stored at − 80 °C were included (41 MSA, 37 CTRL). The samples consisted of both grey and white matter tissue. The brains had been donated to the Brain Bank at Bispebjerg-Frederiksberg Hospital (University Hospital of Copenhagen, Denmark), the MRC London Neurodegenerative Diseases Brain Bank (King’s College London, United Kingdom), or the Netherlands Brain Bank (Royal Netherlands Academy of Arts and Science, Netherlands). For the Danish and Dutch samples, all donors provided informed written consent prior to death. For the English samples, informed written consent was retrieved from donors or their next of kin. Diagnoses were performed by trained medical personal according to the current MSA consensus guidelines [21]. Subsequently, included brains underwent pathological examinations to verify the final diagnosis. Subtype diagnoses were available for 20 patients, and they were divided into an olivopontocerebellar subtype (MSA-C), a striatonigral subtype (MSA-P) or a mixed subtype where neither cerebellar ataxia nor parkinsonism were the dominating feature (MSA mixed). For the remaining patient samples, the sub-diagnoses were unknown. Demographic data are shown in Table 1 and Suppl. Table 1, Online Resource 1. This project was approved by the regional ethical committee of the capital region of Denmark, j.nr. H-16025210. All experiments were conducted in accordance with the Declaration of Helsinki [22].

**Table 1:** Summary of demographic data

**EWAS**							
**Group**	**Brain Bank**		**Sex**		**Age**	**PMI**	
**CTRL**	BBH: 9	KCL: 28	M: 19	F: 18	73.0 ± 10.5	42.0 ± 19.2	–
**MSA**	BBH: 17	KCL: 24	M: 17	F: 24	66.0 ± 5.7	42.0 ± 26.3	–
***P***	0.150		0.496		7.12E-04	0.999	–
**RT-qPCR**							
**Group**	**Brain Bank**		**Sex**		**Age**	**PMI**	**RIN**
**CTRL**	BBH: 10	NBB: 10	M: 8	F: 12	73.5 ± 12.0	28.2 ± 27.4	5.3 ± 0.6
**MSA**	BBH: 16		M: 5	F: 11	64.4 ± 6.0	40.6 ± 20.3	5.1 ± 0.7
***P***	7.58E-04		0.731		6.53E-03	0.128	0.373
**Flow cytometry**							
**Group**	**Brain Bank**		**Sex**		**Age**		
**CTRL**	BBH: 46		M: 18	F: 28	71.9 ± 9.4	–	–
**MSA**	BBH: 24		M: 11	F: 13	62.9 ± 7.9	–	–
***P***	–		0.618		8.46E-05	–	–

Demographic summaries are shown for the cohorts for Illumina MethylationEPIC data (EWAS), RT-qPCR data, and flow cytometric data. Group differences were tested using Fisher’s exact test (sex, origin), or t-tests. Age in years at death is reported; *CTRL* normal, healthy control, *MSA* multiple system atropy, *BBH* Bispebjerg Brain Bank, *KCL* King’s College London Brain Bank, *NBB* Netherlands Brain Bank, *M* male, *F* female, *PMI* Post-mortem interval in hours, *RIN* RNA Integrity Number

### DNA methylation arrays

DNA was isolated from 50 mg brain tissue as described in Online Resource 2. Bisulphite (BS) and oxidative bisulfite (oxBS) treatments were performed using the TrueMethyl Array Kit (CEGX, v. 3.1, March 2017) following the manufacturer’s recommendations. A digestion control was included for all samples. Both sample treatment, and array sample position was randomized in order to eliminate batch effects. In brief, 1 μg gDNA per sample was denatured for 5 min at 37 °C. Then, samples were divided into two fractions for subsequentBS and oxBS treatment. The samples were oxidized (oxBS fraction), converting hydroxymethylated cytosines to formylcytosines, or mock treated (BS fraction) for 10 min at 40 °C. Samples were bisulfite treated for 2 h, and then desulfonated for 5 min before elution. Digestion efficiency was assessed by PCR amplification and gel electrophoresis using the QIAquick PCR Purification Kit (Qiagen; #28104) for DNA clean-up following the manufacturer’s instructions. Amplicon concentrations were measured using the Qubit dsDNA HS Assay Kit (Invitrogen; #Q32854) on a Qubit 2.0 Fluorometer (Life Technologies). One aliquot of purified amplicons was saved for gel analysis. Amplicons were digested using restriction enzymes by incubation at 65 °C for 18 h before denaturation at 80 °C for 20 min. The digested and undigested samples were run on a 2% agarose gel with SYBR Safe (1:10; Invitrogen; #S33102) to assess digestion efficiency. Next, 200 ng of the treated samples were hybridized to Infinium Methylation EPIC BeadChip arrays (Illumina; #WG-317) and imaged on an iScan system (Illumina).

### Bioinformatics and statistics

The bioinformatic analyses were performed in R v. 3.5.0 [23] using *ChAMP* v. 2.13.5 [24]. Data are available at GEO (GSE143157). Data were mapped to *Homo sapiens* GRCh37 unless otherwise noted. Initially, two samples were excluded due to high fraction of failed probes or mismatch between the stated sex versus the predicted sex from the *getSex* function from the *minfi* package [25] (data not shown). For the remaining 78 samples, probes were loaded [25] and filtered [26, 27] based on standard settings yielding 731,661 5mC probes. Samples were normalized using BMIQ [28]. After normalization, we investigated sample variability in our setup. We calculated the intra-assay coefficient of variation to 6.3% (*n* = 2), and the inter-assay coefficient of variance to mean 9.9% (range 6.4–13.8%, *n* = 16). Using hierarchical clustering (Suppl. Fig. 2a, Online Resource 3), we identified a group of outliers consisting of eight MSA and seven CTRL samples. These were not associated with any specific technical or clinical parameters. These samples were removed from downstream analyses, and 63 samples remained. For the 5hmC fraction, methylation levels were calculated as the delta values between the BS and oxBS treated fractions of the samples. Negative values were denoted as NAs, and probes with a fraction of NAs > 0.2 were removed. The remaining NAs were imputed using kNN imputation from the *impute* package [29]. This left us with 405,408 5hmC probes. Following the recommendations by Lunnon et al. [30], we found 62,653 probes with β < 0.046 (the lowest 5th percentile of negative ΔβBS-oxBS across all samples) that were removed in the secondary analyses. Batch effects were investigated using SVD plots [31] (Suppl. Fig. 2b-c, Online Resource 3). No batch effects were identified for the first principal component for any of the fractions (5mC or 5hmC), which accounted for the largest single contribution to the observed variation (Suppl. Fig. 2d-e, Online Resource 3). We calculated the neuronal fraction in our samples as previously described [13] using the *estimateCellCounts* function from the *minfi* package [25], and the *FlowSorted.DLPFC.450 k* package. Differentially methylated probes were identified using *limma* [32] using a linear regression model including age and the neuronal fraction for which the Benjamini-Hochberg method was used to control the False Discovery Rate [33]. Age was included in the model since the MSA patients were significantly younger than the CTRLs (Table 1). Q-Q plots are shown in Suppl. Fig. 2f-g, Online Resource 3. We compared overlapping results with other EWAS studies on brain tissue by considering all our probes with FDR < 0.20, and compared it to available probe/gene lists from four other studies (all FDR < 0.05): Bettencourt et al. (their Suppl. Tables 2.1–2.4) [8], Weber et al. (their Suppl. Table 2) [13], De Jager et al. (their Suppl. Table 2) [34], and Gasparoni et al. (their Suppl. Tables 4, 6, 7, 12, 13, 16, 18) [10]. *Bumphunter* was used for identification of differentially methylated regions or blocks, the model included age and neuronal fraction as for differentially methylated probe analyses [35, 36]. The champ. EpiMod function based on the FEM package was used to identify epigenetic modules based on an agnostic approach using beta values for all probes using CTRL sample data as reference [37]. FANTOM5 [38] enhancer overlaps were evaluated with predefined brain-specific tracks from SlideBase [39]. Chromatin state overlaps were evaluated based on chromHMM [40] analyses from predefined tracks from the NIH Roadmap Epigenomics consortium [41]. Motif enrichment was analysed using *Analysis of Motif Enrichment* from MEME Suite v.5.0.5 [42] against the *HOCOMOCO* Human v. 11 database [43] using 15 bases downstream and upstream from relevant probes mapping to cytosine positions. Q-Q and Manhattan plots were produced with *qqman* v.0.1.4 [44].

### Validation of DNA methylation and hydroxymethylation levels using amplicon sequencing

Validation was performed using the BiSulfite Amplicon Sequencing (BSAS) approach based on the separate amplification of individual regions of interest, followed by tagmentation and next generation sequencing [45] described in Online Resource 2.

### RNA expression analysis

RNA was extracted from 16 MSA and 20 CTRL brain samples of which 15 MSA and 6 CTRL samples overlapped with the samples included in the EWAS analysis. Demographic data for this sub-cohort are shown in Table 1 and Suppl. Table 3, Online Resource 1. RNA was extracted using the miRNeasy Mini Kit (Qiagen; #217004) following the manufacturer’s instructions [20]. The protocol is described in detail in Online Resource 2. Reverse transcription quantitative real-time PCR was performed as earlier described [20] in accordance with the MIQE guidelines [46]. We utilized primers for *AREL1* (PrimerBank ID [47]: 87116667c3, 132 bps) and MHC Class I *HLA*s (covering *HLA*s *A-C* and *E-G*; F: 5-CCTACGACGGCAAGGATTAC-3, R: 5-TGCCAGGTCAGTGTGATCTC-3 [48], 304 bps). Sample cycle threshold (Ct) values were normalised to the expression of the reference genes *UBE2D2*, *TOP1* and *CYC1*, as determined earlier [49], using the geometric mean [50].

### Flow cytometry

Flow cytometric analyses were performed on peripheral blood mononuclear cells from an independent cohort of 24 MSA patients and 46 CTRLs following a previously described procedure [51]. Patients with a probable MSA-P or MSA-C diagnosis were included. Four of these patients died during the course of the study and had agreed to donate their brains to the brain bank, and their diagnoses were pathologically validated. None of the patient samples overlapped with the DNA or RNA brain samples used in this study. Demographic data are shown in Table 1 and Suppl. Table 4, Online Resource 1.

### Single-cell RNA expression

We investigated expression of relevant targets in public single-cell RNA data datasets from the BRAIN Initiative Cell Census Network (BICCN, RRID:SCR_015820; https://biccn.org/) uploaded to the Neuroscience Multi-omic Archive (NeMOarchive, RRID:SCR_016152; https://nemoarchive.org/data/). Data originated from the human primary motor cortex prepared using Smart-seqV4 reagents. Data were available for 11,577 cells which was prepared for analysis using *PAGODA2* (https://github.com/hms-dbmi/pagoda2) and *Conos* [52] in R v. 3.5.0 [23].

## Results

### Differentially methylated probes in MSA patients

We analysed genome-wide DNA methylation and hydroxymethylation profiles using an array-based approach in the prefrontal cortex of 41 MSA patients and 37 CTRLs. We identified differentially methylated probes using a linear regression model that included age as well as an estimation of the fraction of neuronal cells. We estimated the fraction of neurons in our samples as previously described [13]. The neuronal fraction did not differ between groups (Wilcoxon’s non-parametric t-test, W = 514, *P* = 0.800).

**Fig. 1:**
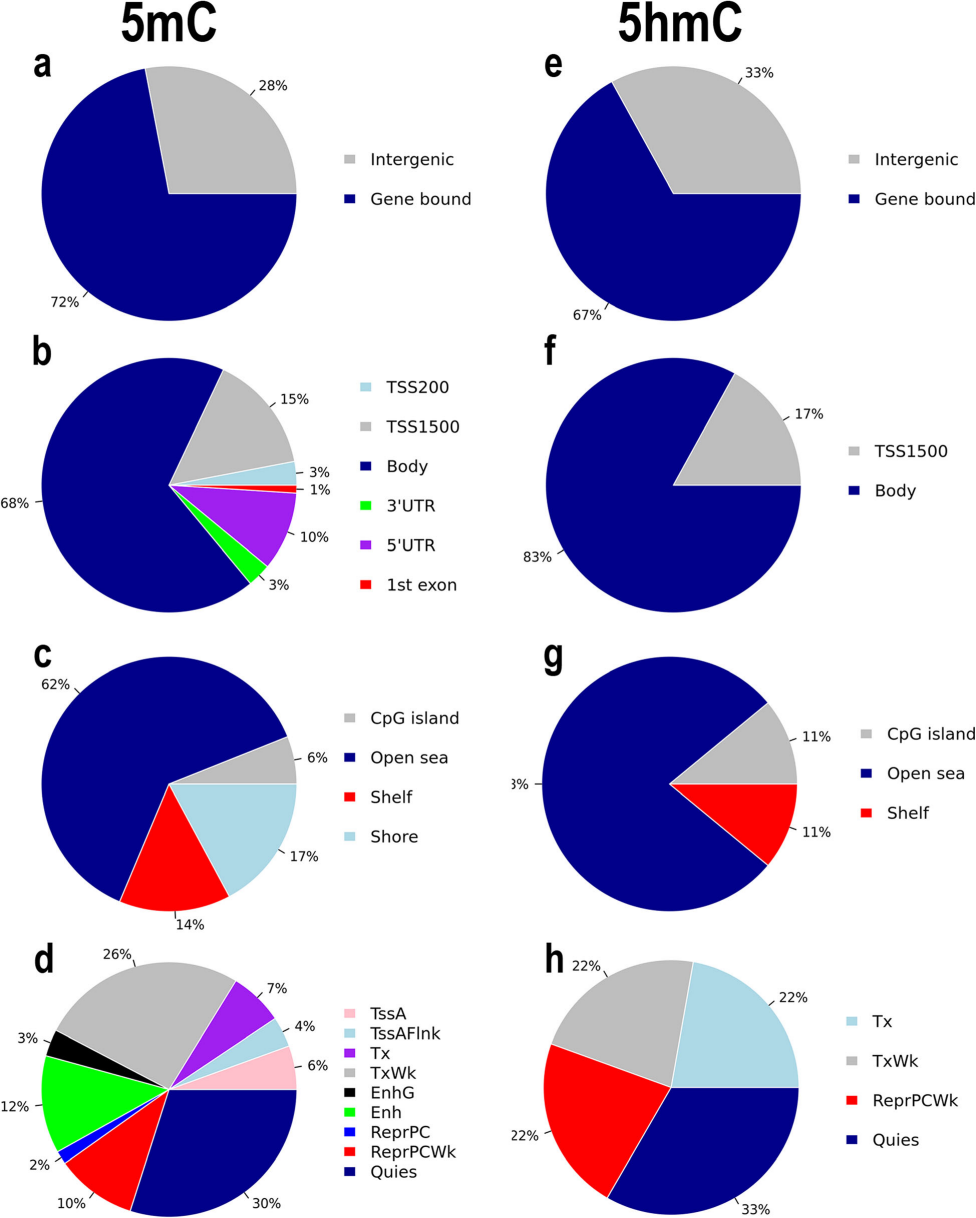
CpG probe distribution. **a-h** Locations of differentially methylated probes in multiple system atrophy (MSA) patients and normal, healthy controls (CTRLs) for the 5-methylcytosine (5mC; **a**-**d**) or 5-hydroxymethylcytosine (5hmC; **e**-**h**) fractions at FDR < 0.20. Probe distributions are shown for **a**,**e** intergenic or gene bound genomic areas; **b**,**f** 200 or 1500 bases upstream of transcription start site (TSS), in gene bodies, at the 3′ or 5′ untranslated region (UTR), or in the first exon; **c**,**g** in CpG island, shelf or shore areas, or in open sea areas; **d**,**h** and the CpG positions according to chromHMM tracks for the dorsolateral prefrontal cortex (TssA: active TSS; TssAFlnk: flanking TssA; Tx: strong transcription; TxWk: weak transcription; EnhG: genic enhancer; Enh: enhancer; ReprPC: repressed PolyComb; ReprPCWk: weak ReprPC; Quies: quiescent or low transcription)

For the 5mC fraction, 731,661 probes remained after filtering while for the 5hmC fraction, 405,408 probes remained. Using a stringent Benjamini-Hochberg correction for multiple comparisons (FDR < 0.05), we identified five differentially methylated probes in MSA compared with CTRLs in the 5mC fraction (Table 2). Of these five probes, two mapped to gene bodies in *AREL1* or *KTN1* genes whereas the other probes mapped to intergenic regions. When we assessed CpGs with a relaxed correction for multiple testing (FDR < 0.20), 234 CpGs for the 5mC fraction (Fig. 1a-d) and nine CpGs for the 5hmC fraction (Fig. 1e-h) remained (Suppl. Table 5, Online Resource 1). Considering these probes, for the 5mC fraction we identified an enrichment of CpGs in CpG island shelf regions (hypergeometric test, *P* = 2.77E-05, 33 probes). For the 5hmC fraction, enrichment was identified for CpGs in CpG open sea regions (hypergeometric test, *P* = 1.66E-03, 7 probes). Further, we investigated overlap with enhancer regions, and motif enrichment for CpGs in TSS200 or TSS1500 regions. By integration with chromHMM data for chromatin states of the dorsolateral prefrontal cortex, we identified 42.3% of the listed CpGs to reside in Transcription Start Site (TSS)-related regions or in transcription-enriched regions for 5mC probes (Fig. 1d). No CpGs resided in brain-specific FANTOM5 enhancer regions. We identified two enriched motifs for 5mC methylation levels with adj. *P* < 0.05 that all bind GC-rich regions on the DNA: One motif related to HINFP activity (adj. *P* = 1.57E-02), and one motif related to ZIC3 activity (adj. *P* = 2.11E-02).

**Table 2:** Differentially Methylated Probes

Fraction	Probe ID	Chr	Position	Gene	Gene feature	CpG island	Δβ (%)	***P***	Adj. ***P***	chromHMM	Function
5mC	cg08753407	14	75,151,317	*AREL1*	Body	Open sea	−0.09	1.47E-07	3.60E-02	5_TxWk	Ubiquitination and antigen presentation
5mC	cg03452759	2	31,467,215		IGR	Open sea	0.03	1.91E-07	3.60E-02	15_Quies	
5mC	cg24646067	5	87,812,057		IGR	Open sea	−0.08	2.00E-07	3.60E-02	15_Quies	
5mC	cg27312312	14	56,046,001	*KTN1*	TSS1500	Shore	0.04	2.08E-07	3.60E-02	1_TssA	Microtubulue-associated protein
5mC	cg16096172	6	46,924,482		IGR	Open sea	0.03	2.46E-07	3.60E-02	15_Quies	

Differentially methylated probes (FDR < 0.05) in the 5-methylcytosine (5mC) fraction. *Probe ID* Illumina probe ID, *Chr* chromosome, *Position* chromosomal position, *chromHMM* ChromHMM [40] data for the dorsolateral prefrontal cortex; *5_TxWk* weak transcription activity, *15_Quies* quiescent transcription, *1_TssA* active TSS

### *AREL1* presents a shift from cytosine methylation to cytosine hydroxymethylation

**Fig. 2:**
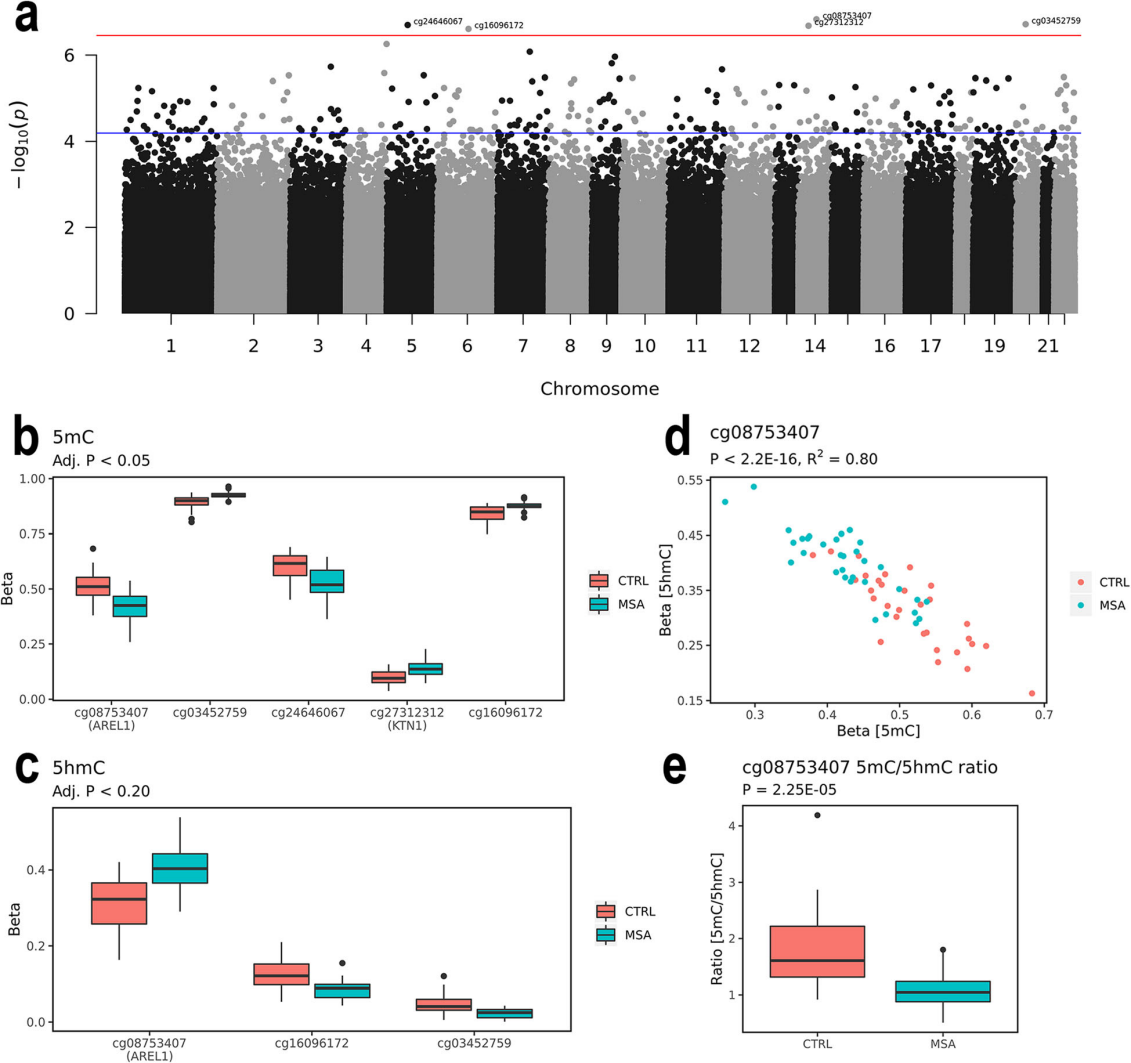
EWAS results. **a** Manhattan plot showing the distribution of *P* values for differences between multiple system atrophy (MSA) patients and normal controls (CTRLs) along the chromosomes. The horizontal red line indicates the adj. *P* value cut-off, the horizontal blue indicates cut-off for FDR < 0.20. **b-c** Box plots showing **b** the 5-methylcytosine (5mC) or **c** 5-hydroxymethylcytosine (5hmC) levels of MSA patients and CTRLs. Boxes show 1st (Q1) to 3rd (Q3) quartile of data, the horizontal line shows the median, and the whiskers show Q1-inter quartile range (IQR; lower whisker) or Q3 + IQR (upper whisker). Outliers are shown with dots. **d** Correlation of 5mC and 5hmC beta values. MSA are green, CTRLs red. **e** Box plot showing the 5mC/5hmC ratio

Of the five significant probes with FDR < 0.05 (Fig. 2a, b), two probes showed a mean difference > 5% with one probe mapping to *AREL1* (cg08753407, change (Δ) in methylation (β) = − 9.1%, *P* = 1.47E-07; Fig. 2b) belonging to the E3 ubiquitin ligase family [53] necessary for antigen presentation [54]. Hence, this change in methylation indicates an immune activation in MSA patients. Consistently, the probe mapping to *AREL1* was also the most significant probe in the 5hmC fraction showing an increase in MSA patients (Δβ = 8.5%, *P* = 2.69E-07; Fig. 2c) although without passing the correction for multiple testing (Suppl. Table 5, Online Resource 1). When we removed probes with small ΔβBS-oxBS values that might not be reliably detected [30], we confirmed cg08753407 on *AREL1* to be the most significant probe (*P* = 2.28E-07, Suppl. Table 5, Online Resource 1). Additionally, the 5mC levels correlated with the 5hmC levels (Pearson’s correlation, *P* < 2.2E-16, R^2^ = 0.80; Fig. 2d), and the 5mC/5hmC ratio differed between groups (Welch’s t-test, t = 4.77, *P* = 2.25E-05; Fig. 2e). The other significant probe with mean difference > 5% mapped to an intergenic region (IGR) on chromosome 5 (cg24646067, Δβ = − 0.08%, *P* = 2.00E-07; Table 2) 148 Mb upstream of the non-coding gene *LINC00461*.

Subtype diagnoses were available for 17 of our 33 MSA patients, nine MSA-C, six MSA-P and two MSA mixed. We investigated differentially methylated probes between MSA-C and MSA-P patients, however, no probes passed a relaxed threshold for multiple correction (FDR < 0.20; Suppl. Table 6, Online Resource 1). Additionally, methylation of the five significant probes in the 5mC fraction did not differ between subtypes (Student’s t-test, *P* > 0.05) in concordance with the assumption that the two subtypes represent the same disease.

We applied an agnostic approach to investigate methylation changes in small genomic regions spanning 100 s to 1000s of bases (DMRs) as well as large genomic regions spanning millions of bases (blocks). For the 5mC fraction, ten DMRs (Suppl. Table 7, Online Resource 1) were identified. Additionally, we identified one block (chr6:64,308,555-64,423,797; *P* = 3.52E-06) covering *PHF3*. The DMRs covered regions with genes having several different functions, including neuronal signalling (*CHRNE*, *NCS1*). Additionally, the block spanned *PHF3* is involved in glioblastoma development.

In order to perspective our results to other EWAS studies on brain tissue from neurodegenerative diseases, we investigated overlaps for our probes with an adj. *P* < 0.20 and four other studies: one MSA study [8], one PSP study [13], and two AD studies [10, 34]. In total, we investigated 2181 unique probes and 1239 unique genes from these studies. For the 5mC fraction, we identified overlaps for two probes and 20 genes whereas one gene overlapped in the 5hmC fraction (Suppl. Table 8, Online Resource 1). Four genes were shared with the other EWAS on tissue from MSA patients, eight genes and one probe were shared with the EWAS on PSP tissue, while 14 genes and one probe were shared with the AD studies. No probe or gene were shared between all studies. Fourteen probes with FDR < 0.20 on the overlapping genes were present in both the 5mC and the 5hmC fraction, and Δβ changes were oppositely corresponding to each other (Suppl. Fig. 3, Online Resource 3). The functions of these genes are related to the extracellular matrix (*COL23A1*, *LTBP3*) and the immune system (*PTPRN2*, *CYFIP1*) while *TIMP2* falls in both these categories. Furthermore, we investigated total methylation levels in order to compare our results directly to the study by Bettencourt et al. [8]. Specifically, Bettencourt et al. highlights probes on *HIP1*, *LMAN2*, and *MOBP* genes, however, in our setup the most significant probes on *HIP1* and *LMAN2* genes were only nominally significant (cg08710628 on *HIP1*, *P* = 0.003; cg05408837 on *LMAN2*, *P* = 0.008), whereas no probes on *MOBP* were significant. We investigated overlap with the 157 probes highlighted by Bettencourt et al. from their cross-region analysis. Seven probes from the current study had a nominally significant *P* < 0.05, however, no probes passed correction for multiple testing (Suppl. Table 5, Online Resource 1)*.*

We validated the validity of the results from the MethylationEPIC array using high-throughput amplicon sequencing of 19 CpG methylation levels (ten 5mC, nine 5hmC). Our approach allowed us to assess methylation changes in the regions surrounding CpGs of interest. In total, 16 of the 19 CpGs showed methylation changes in the same direction as the array. Furthermore, we identified nominally significant methylation changes on surrounding CpG positions for several of the investigated loci, including several positions on *HLA-A*, *HLA-F*, and *ZIC4* (Suppl. Table 10, Online Resource 1)*.*

### *SNCA* and other disease-related genes show no significant differential methylation

We investigated differentially methylated CpGs on ten genes that have been deemed important to MSA. These included *COQ2*, *ELOVL7*, *GBA*, *LRRK2*, *MAPT*, *PARK2*, *PARK7*, *PINK1*, *SLC1A4*, and *SNCA* (Suppl. Table 11, Online Resource 1). For the 5mC fraction this included 442 probes, whereas it included 243 probes for the 5hmC fraction. Although we identified several probes that were nominally significant, no probes passed the correction for multiple comparisons.

### Epigenetic modules on inflammation-related genes are changed in MSA patients

We continued our agnostic approach by investigation of functional epigenetic modules. All modules are summarized in Table 3. When looking further into both 5mC and 5hmC fractions separately, 11 modules were identified that are involved in biological functions such as cellular functions (5mC: *DNMT3B*, *VAMP8*; 5hmC: *GRK2*, *SNRPB*). Additionally, in support of immune system involvement in MSA, four modules were related to inflammation (5mC: *FCER1G*, *TNF*; 5hmC: *ITGA4*, *ZBTB16*; Suppl. Fig. 4–5, Online Resource 3).

**Table 3:** Functional Epigenetic Modules

Fraction	Seed	***P*** value	Function
**5mC**	*FCER1G*	0.006	Antibody-binding receptor
	*ELN*	0.011	Elastic fiber formation
	*MDFI*	0.012	Repression of myogenesis
	*TNF*	0.035	Cytokine
	*DNMT3B*	0.038	DNA methyltransferase
	*VAMP8*	0.047	Synaptic vesicle function
**5hmC**	*GRK2*	0.004	GPCR
	*SNRPB*	0.008	Ribonucleoprotein
	*SSTR3*	0.009	Somatostatin receptor
	*ITGA4*	0.019	Lymphocyte homing receptor
	*ZBTB16*	0.023	Zinc finger transcription factor

Summarization of identified functional epigenetic modules calculated for both the 5-methylcytosine (5mC) and the 5-hydroxymethylcytosine (5hmC) fractions. Seed: Center gene of module

### Increased *AREL1* and MHC class I *HLA* gene expression in MSA brains

**Fig. 3:**
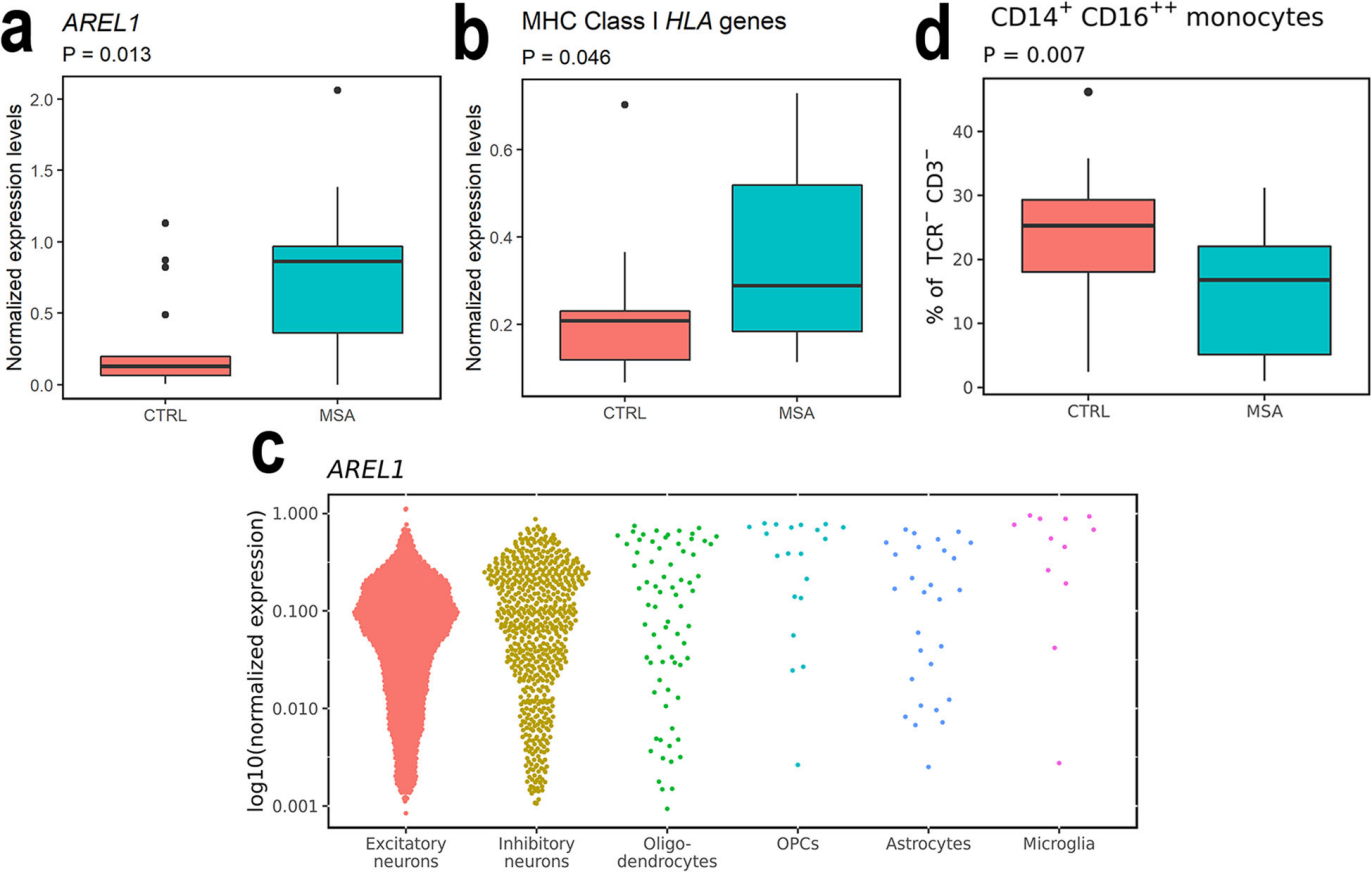
Expression of *AREL1*, MHC Class I *HLA*s and blood CD14^+^CD16^++^ monocyte changes. **a**-**b** RT-qPCR experiments quantifying *AREL1* (**a**) or MHC Class I *HLA*s (*HLA-A*, *−B*, −*C*, −*E*, −*F*, −*G*; **b**). Data were normalized to the geometric mean of three reference genes *UBE2D2*, *RPL13*, and *TOP1* [49]. **c** Dot plot showing normalized expression of *AREL1* in oligodendrocytes, astrocytes, inhibitory neurons, excitatory neurons, oligodendrocyte precursor cells (OPCs), and microglia cells from five healthy human frontal cortical samples. Data for 11,577 cells are shown. **d** Levels of non-classical CD14^+^CD16^++^ monocytes in peripheral blood mononuclear cells as the fraction of TCR^−^CD3^−^ cells. Boxes show 1st (Q1) to 3rd (Q3) quartile of data, the horizontal line shows the median, and the whiskers show Q1-inter quartile range (IQR; lower whisker) or Q3 + IQR (upper whisker). Outliers are shown with dots

We proceeded to investigate *AREL1* gene expression levels in a sub-cohort of our samples (16 MSA, 20 CTRL; Table 1, Suppl. Table 3, Online Resource 1). We observed increased expression of *AREL1* in the prefrontal cortex of MSA patients compared with CTRLs (Mann-Whitney t-test, U = 67, *P* = 0.013; Fig. 3a). Normalized *AREL1* gene expression did not correlate with neither 5mC nor 5hmC levels (*P* > 0.05, data not shown). Additionally, *AREL1* expression did not correlate with age, sex, PMI or RIN (*P* > 0.05). Based on the involvement of *AREL1* in MHC class I antigen presentation, we decided to investigate whether we could detect increased *HLA* expression in brains of the MSA patients by quantification of joint mRNA expression of MHC Class I (*HLA*s *A-C* and *E-G*). We observed an increased MHC Class I *HLA* expression in MSA patients compared with CTRLs (Welch’s t-test, t = 2.777, *P* = 0.013; Fig. 3b). Furthermore, MHC class I gene expression was not correlated with age, sex, PMI or RIN (*P* > 0.05).

### *AREL1* is mostly expressed in neurons

We investigated expression of *AREL1* in single-cell RNA-sequencing data from healthy human frontal cortex samples. We identified expression of *AREL1* in all the investigated cell types, including excitatory and inhibitory neurons, oligodendrocytes, oligodendrocyte precursor cells, astrocytes, and microglia. Neurons, especially excitatory neurons, were the cell type with the highest fraction of cells expressing *AREL1* (Fig. 3c).

### Differences in peripheral blood mononuclear cell composition in MSA patients

In order to support our observations of an immune activation in MSA patients, we investigated the peripheral immune system. We wanted to confirm that immune alterations are present during the disease course and not just during disease end-stage. Using blood samples from a new patient cohort (24 MSA, 46 CTRL), we investigated the composition of peripheral blood mononuclear cells (PBMCs). We identified a decrease in the fraction of non-classical CD14 + CD16++ monocytes (F(2,67) = 4.235, *P* = 0.019, R^2^ = 0.112) determined by group (*P* = 0.007; Fig. 3d). This finding was neither correlated to disease duration nor Hoehn & Yahr staging (Spearman’s correlation, *P >* 0.05). We did not see any difference in activated CD45RA^+^ non-classical monocytes (F(2,67) = 1.506, *P* = 0.229, R^2^ = 0.043). Further, we did not observe any differences in CD4^+^ or CD8^+^ T cell composition, nor in CD56^+^ or CD57^+^ NK cell fractions (Suppl. Table 12, Online Resource 1).

## Discussion

In the present study, we evaluated epigenetic modifications in brains of MSA patients at both methylated and hydroxymethylated cytosines in the DNA. Most importantly, we identified a shift from cytosine methylation towards hydroxymethylation, a modification commonly associated with increased gene expression activity, at the cg08753407 probe mapping to the *AREL1* gene in MSA patients. The *AREL1* gene codes for an E3 ubiquitin ligase involved in protein ubiquitination and degradation [53]. Further, E3 ubiquitin ligases mark proteins for degradation in the proteasome which is necessary for antigen presentation through MHC Class I complexes (reviewed by Loureiro & Ploegh [54]). This methylation shift on *AREL1* was further illustrated by a change in the 5mC/5hmC ratio between sample groups, which would not be detectable by analysing total DNA methylation levels using standard bisulfite treatment. The altered ratio accommodated an increase in *AREL1* gene expression in MSA patients, which is accompanied by increased expression of MHC Class I *HLA* genes. In the blood, we found the fraction of non-classical CD14^+^CD16^++^ monocytes to be decreased in MSA patients, whereas no differences were observed for the T cell or NK cell fractions. Collectively, our results support that MSA patients present an active neuroimmune response through increased antigen presentation, which is further reflected by a change in the composition of blood immune cells that does not involve neither T cells nor NK cells.

Together with these findings, we also identified several changes related to immune system responses in the MSA brains. The identification of altered epigenetic modules further support the involvement of innate and adaptive immune compartments in MSA. The most prominent modules that seems to be affected in MSA, are related to the lymphocyte homing receptor (*ITGA4*) [55], the antibody-binding receptor *FCER1G* [56], and the cytokine *TNF* [57], which has been previously investigated in MSA patients. To characterize whether the observed differences in gene methylation are functionally relevant, we investigated gene expression of *HLA* molecules. We identified a joint increase in expression of *HLA*s *A-C* and *E-G* genes thereby showing a possible link between methylation changes and antigen presentation. Our current observations on gene methylation changes encourage further investigations into the possible involvement of these genes in the pathology of MSA.

Finally, supporting the observations in brain tissue, there seems to be a systemic immune dysregulation in MSA as shown by the results on peripheral immune cells in a novel cohort of MSA patients and controls. We did not see disease-related differences in T cell levels in blood, but we identified a decreased fraction of non-classical CD14^+^CD16^++^ monocytes in MSA patients. Only classical monocyte levels have earlier been reported for a small cohort of MSA patients, however, the authors failed to identify differences for the MSA group compared with controls [58]. The exact role of non-classical monocytes in chronic diseases is not clear, but they are considered as anti-inflammatory, as they maintain vascular homeostasis [59]. The decrease in non-classical monocytes in MSA observed by us resembles what has been described for hereditary diffuse leukoencephalopathy with spheroids (HDLS) [60]. HDLS is an autosomal dominant white matter disorder [61] that is accompanied by parkinsonian features [62] and may present with Lewy bodies inclusions [63]. HDLS is caused by mutations in *CSF1R,* the receptor for colony-stimulating-factor (CSF), which is a growth factor for microglia, monocytes and macrophages [64].

Of interest, both MSA [65–67] and HDLS [68] share pathological astro- and microgliosis. In a previous study we found that MSA patients had lower protein levels of G-CSF, a growth factor belonging to the same family as CSF, in the prefrontal cortex of MSA brains [20]. These observations seem to support each other, as the observed decline in non-classical monocytes observed in blood of MSA patients is probably associated with the neuroinflammatory state of the patients. Additionally, in support of an increased inflammatory state in MSA, our laboratory has previously shown aberrant gene expression of Toll-like receptors in different brain areas of MSA patients [69]. Expression of these receptors is induced in response to infection as well as cell death [70]. Furthermore, the recent EWAS from Bettencourt et al. on MSA patients identified reduced total methylation on a probe in the TSS1500 region of *IL2RA* in MSA patients with the mixed subtype compared to normal controls [8]. Taken together, these results all support an involvement of an innate immune response in MSA.

We investigated whether methylation changes related to subtype diagnoses could be detected, however, we did not observe any MSA subtype-specific methylation. In contrast, Bettencourt et al. identified several subtype-specific changes [8]. In general, they observed the strongest effect for the MSA-C subtype. However, Bettencourt et al. investigated different brain tissues including the cerebellum, an area that is severely affected in MSA-C patients [71]. Hence, our results do not support subtype-specific changes in the prefrontal cortex, which was the area of interest in the present study. Further, we investigated overlaps between our results with other EWAS studies on brain tissue. Although several overlaps were identified, the involved biological functions were equivocal (Suppl. Table 8, Online Resource 1). Several overlapping genes are related to inflammatory processes (*TIMP2*, *CYFIP1*, *PTPRN2*, *CUX1*) whereas the remaining overlaps are related to different cellular processes. The apparent inconsistency between studies and diseases could be a result of distinct pleiotropic epigenetic processes in the brain disorders as well as differences in the experimental set-up and analysed tissues. Future comparative studies are encouraged to shed light of disease-dependent and independent epigenetic traits in neurodegenerative diseases.

Our analyses also revealed possible effects unrelated to inflammation. On brain material from the prefrontal cortex, we identified a significant probe in the TSS1500 region of *KTN1* (cg27312312, Δβ = 3.9%, *P* = 2.08E-07). *KTN1* is a gene coding for kinesin, a protein involved in intracellular vesicle transportation and related to cytoskeletal signalling and Rho GTPase signalling. Interestingly, a meta-analysis of GWAS studies found this gene to be associated with PD [72] suggesting a possible overlap in disease processes between these related diseases. Furthermore, Bettencourt et al. similarly identified Rho GTPase signalling to be involved in MSA pathology [8]. Additionally, the remaining three probes all mapped to IGRs. The probe closest to a gene was cg16096172 on chromosome 6 upstream of *ADGRF5*. Whether methylation changes on *KTN1* or the IGR probes are biologically relevant to MSA pathology remains to be elucidated.

The recent publication by Bettencourt et al. was the first to report on epigenome methylation changes in MSA. In opposition to our study, they employed a region-wise comparison of total methylation changes identifying several significantly different probes and regions in samples from MSA patients. When comparing their results to our most significant probes, four gene overlaps were identified, one of them involved in the immune system (*CYFIP1*) and others involved in extracellular matrix regulation (*COL23A1*, *CTBP3*). Furthermore, in the present study we investigated both 5mC and 5hmC levels whereas Bettencourt et al. investigated only total methylation levels. We compared total methylation levels from our study on probes mapping to relevant genes identified by Bettencourt et al., however, no apparent overlaps were found. Furthermore, whereas 141 probes overlapped with the 157 probes identified in the cross-region analysis by Bettencourt et al., only seven probes had a nominal *P* < 0.05 while none of them had an adj. *P* < 0.05. The differences between the two studies may be explained in the selection of the studied tissue. Whereas Bettencourt et al. investigated white matter samples from different areas across the brain, we investigated samples that included both white and grey matter. When considering recent technological developments for single-cell assessment of epigenetic changes, it would be relevant in future studies to investigate the epigenetic contribution to changes in brain samples from MSA patients at the single-cell level.

In the current study, the following limitations must be considered. First, although we identified methylation changes in the prefrontal cortex of MSA patients, larger effects may be found in other brain region as demonstrated by Bettencourt et al. Indeed, a recent study screened for epigenetic changes in different brain areas in PD and MSA patients which showed a global increase of 5hmC intensity in the white matter of the cerebellum in both PD and MSA patients [19], while no difference in methylation levels was observed in the neocortex between PD patients and controls. Therefore, a careful evaluation of the area of interest prior to the initiation of novel epigenetic studies for MSA should be performed. In the current study we aimed to investigate a brain area previously shown to be affected in MSA [20, 65]. This approach was chosen in order to model epigenetic changes occurring at early stages of disease development and prior to massive cell death. Therefore, it was not in the scope of the current study to compare regional 5mC or 5hmC differences. Second, in the current study we investigated methylation changes on bulk brain samples since we hypothesized epigenetic changes to be large and to some degree cell independent. However, a recent post-mortem study in AD [10] showed the importance of cell stratification for investigating epigenetic changes in neurodegenerative diseases in order to detect changes using small sample numbers, even smaller than what was employed in the current study. We sought to approach this shortcoming by estimating the fraction of neuronal and glial cells in our samples, which we included in our regression model. Nevertheless, for future studies we encourage a priori isolation of the cell populations of interest prior to the epigenetic analyses. Third, since MSA patients have a shorter life expectancy than healthy individuals [3], MSA patients were on average 6.90 years younger than CTRLs in our setup. Since global hypomethylation occurs during aging [73], we included age as a covariant in our regression model thereby rejecting identification of significant probes affected by aging. Fourth, we investigated post-mortem tissue and therefore we cannot establish causality between our findings and development of MSA.

Conversely, our study set-up holds several strengths. First, and most importantly, we investigated hydroxymethylation levels which has not earlier been investigated in MSA. By extrapolating changes identified in the 5mC fraction to the 5hmC fraction we showed strongly correlated and concordant changes between methylation states underlining the biological validity of our results. Second, we applied a stringent bioinformatical approach where we first identified and removed outliers to reduce noise in our data. Third, our patient samples were all diagnosed by trained clinical personal, and the samples originated from different centres in different countries thus minimizing a possible regional bias. Additionally, all samples underwent pathological investigations to validate the diagnosis. Fourth, we performed a technical validation of the bead arrays as well as biological validation using RT-qPCR to investigate the effect of methylation changes on gene expression levels. Finally, we sought to determine the effects of our results on brain tissue by investigation of PBMC changes in samples from a novel patient cohort. Although we identify an atypical change in non-classical CD14^+^CD16^++^ monocytes that we cannot relate directly to our results on brain tissue, future evaluations of these biological differences may further identify the exact molecular aberrancies in MSA patients explaining our results.

## Conclusions

To conclude, we identified several CpGs with genome-wide significance including a shift from 5mC to 5hmC methylation of the cg08753407 probe and associated gene expression changes of *AREL1* in MSA patients*,* a gene related to antigen presentation. Further, these results were accompanied by increased gene expression of MHC Class I *HLA*s further implicating antigen presentation as a disease factor in MSA. Lastly, we saw a decrease in non-classical CD14^+^CD16^++^ monocytes in blood of MSA patients. Taken together, our results provide an epigenetic link between MSA and the immune processes in MSA patients. Not only do our result increase the knowledge about disease processes in MSA, they may also pave the way for immunomodulatory approaches to diagnose, treat, or prevent the onset of MSA.

## Supplementary information

**Additional file 1.** Online Resource 1: Supplementary Tables.

**Additional file 2.** Online Resource 2: Material and Methods.

**Additional file 3.** Online Resource 3: Supplementary Figures.

## Data Availability

Normalized and raw BeadChip Array data have been deposited in NCBI Gene Expression Omnibus (GEO) with the accession code GSE143157. All other data are available within the paper and its associated supplementary material or upon reasonable request from the corresponding authors.
