# Supplementary material for: Epigenetic modulation of AREL1 and increased HLA expression in brains of multiple system atrophy patients
Source: Acta Neuropathol Commun. 2020 Mar 9;8:29. doi: 10.1186/s40478-020-00908-7 (PMC7063795; doi:10.1186/s40478-020-00908-7)
Supplement: Supplementary file 2 — Additional file 2. Online Resource 2: Material and Methods. [file 40478_2020_908_MOESM2_ESM.docx]

**Epigenetic modulation of *AREL1* and increased *HLA* expression in brains of Multiple system atrophy patients**

Rasmus Rydbirk^1,2,3,a^, Jonas Folke^1,3^, Florence Busato^2^, Elodie Roché^2^, Alisha Shahzad Chauhan^1,3^, Annemette Løkkegaard^4,5^, Anne-Mette Hejl^4^, Matthias Bode^6^, Morten Blaabjerg^6,7^, Mette Møller^8^, Erik Hvid Danielsen^8^, Tomasz Brudek^1,3^, Bente Pakkenberg^1,5^, Jorg Tost^2,b^, Susana Aznar^1,3,b,^*

^1^ Research Laboratory for Stereology and Neuroscience, Bispebjerg-Frederiksberg Hospital, University Hospital of Copenhagen, Nielsine Nielsens Vej 6B, DK-2400, Copenhagen, Denmark

^2^ Laboratory for Epigenetics and Environment, Centre National de Recherche en Génomique Humaine, CEA-Institut de Biologie Francois Jacob, 2 rue Gaston Crémieux, FR-91000 Evry, France

^3^ Copenhagen Centre for Translational Research, Bispebjerg-Frederiksberg Hospital, University Hospital of Copenhagen, Nielsine Nielsens Vej 4B, Copenhagen, Denmark

^4^ Department of Neurology, Bispebjerg-Frederiksberg Hospital, University Hospital of Copenhagen, Ebba Lunds Vej 44, DK-2400, Copenhagen, Denmark.

^5^ Institute of Clinical Medicine, Faculty of Health, University of Copenhagen, Blegdamsvej 3B, DK-2200 Copenhagen, Denmark

^6^ Department of Neurology, Odense University Hospital, J.B. Winsløws Vej 4, DK-5000 Odense, Denmark.

^7^ Department of Clinical Research, University of Southern Denmark, Odense, Denmark.

^8^ Department of Neurology, Aarhus University Hospital, Aarhus, Denmark.

^a^ Present address: Biotech Research and Innovation Centre, Faculty of Health, University of Copenhagen, Denmark

^b^ Joint senior authorship

* Corresponding author:

Susana Aznar, PhD

Research Laboratory for Stereology and Neuroscience

Bispebjerg-Frederiksberg Hospital, University Hospital of Copenhagen

Nielsine Nielsens Vej 6B, stair 11B, 2nd floor

DK-2400, Copenhagen, Denmark

E-mail: [susana.aznar.kleijn@regionh.dk](mailto:susana.aznar.kleijn@regionh.dk)

# Materials and methods

## DNA extraction

Genomic DNA (gDNA) was extracted from 50 mg tissue incubated overnight at 50 °C in 500 µl digestion buffer (100 mM NaCl (J.T. Baker; #0277), 1:50 (v/v) Tris-HCl (RegionH Apoteket; #851426), 25 mM EDTA (Sigma-Aldrich; #9884), 0.75% SDS (Sigma-Aldrich; #L3771) in nuclease-free H_2_O (Sigma-Aldrich, W4502)) containing 400 µg proteinase K (Roche; #3115836001). Phase separation was performed using 400 µl phenol:chloroform:isoamyl-OH (25:24:1; Invitrogen; #15593031) and 50 µl nuclease-free H_2_O with gentle mixing before centrifugation at 4 °C for 5 min at 15,000 x *g*. gDNA was pelleted with 0.1 volume of 3M NaOAc (Fluka Chemie GmbH; #71183) and 2 volumes of ice cold absolute EtOH (Sigma-Aldrich; #51976) by incubation at -18 °C for 1 h followed by centrifugation at 4 °C for 10 min at 12,000 x *g*, before washing inice cold 70% EtOH in nuclease-free H_2_O (v/v). Concentrations were measured using the Quant-iT PicoGreen dsDNA Assay Kit (Invitrogen; #P11496) following the manufacturer’s instructions.

## Validation of DNA methylation and hydroxymethylation levels using amplicon sequencing

PCR primers were designed with MethPrimer[1]. Primers are provided in Suppl. Table 2, Online Resource 1. Amplicon primers did not overlap with any CpGs or single nucleotide positions. 1 µg of genomic DNA was treated with TrueMethyl® oxBS Module (NuGen, #0414-32) following the manufacturer’s instructions, which replaced the corresponding CEGX kits. Sample were divided into two fractions, a BS and an oxBS fraction. PCRs were performed on BS and oxBS treated samples using Phusion U Hot Start PCR Master Mix (Thermo Fisher Scientific, #F533L) with 2X of Phusion Master Mix and 0.2 µM of each primer. Cycling consisted of a pre-heating at 98 °C for 15 min, then 35 cycles of 30 s at 98 °C, 30 s at the optimal annealing temperature, 30 s at 72 °C and a final extension step at 72 °C for 7 min. After bead-based purification, PCRs were quantified using a LabChip GX Analyser (Perkin-Elmer) and then pooled by individual (one BS pool and one oxBS pool per individual) at an equimolar ratio. 5 µL of each pool was tagmentated and amplified using the Nextera XT DNA Sample Preparation Kit (Illumina, #FC-131-1096) and Nextera XT Index Kit (Illumina, #FC-131-1002) according to the manufacturer’s instructions. After bead-based purification, libraries were again analysed on a LabChip GX Analyser (Perkin-Elmer) to determine the average size of the fragments. Concentrations were measured by qPCR using the KAPA Library Quantification Kit (Roche, #07960140001). The final concentration was calculated as raw qPCR concentration x 452/size average. Libraries with different sample indexes (96) were combined at equimolar ratios resulting in one BS pool containing all PCRs of all BS treated samples and one pool containing all PCRs of all indexed oxBS samples and sequenced on a MiSeq instrument (Illumina) in paired-end modus with a 2 x 75 bp read length.

### Data analysis for amplicon sequencing

Adapters were trimmed from raw sequences using trimmomatic1 v. 0.32 trimming bases with quality under 30 and sequences with less than 50 bases between adapters. Trimmed sequences were mapped to the GRCh37 using bismark v. 0.19.0. Methylation levels at CpG sites mapping to the regions of interest were obtained using in-house R scripts. CpGs were included in the analysis if more than 30 read counts for the respective CpG were available. 5hmC levels were calculated by subtracting 5mC levels (oxBS) from total methylation levels (BS). 5hmC values below 0 were treated as 0. Total methylation, mean 5mC and 5hmC levels were calculated for patient and control groups, as well as the methylation differences between the two groups. DNA methylation differences were assessed using a Wilcoxon test with *P*<0.05 considered as significant.

## RNA Extraction

RNA was extracted using the miRNeasy Mini Kit (Qiagen; #217004) following the manufacturer’s instructions[2]. All reagents were included in the kit unless otherwise noted. All reagents were included in the kit unless otherwise noted. In short, app. 50 mg brain tissue was extracted from frozen human brains stored at -80 °C and homogenized by pipetting in lysis reagent. Samples were phase separated using chloroform (JT Baker, # 01878410109) before on-column DNase treatment. RNA was eluted in nuclease-free water. Samples were aliquoted and immediately stored at -80 °C. RNA integrity was assessed using the RNA 6000 Nano Kit (Agilent Technologies; #5067-1511) following the manufacturer’s instructions on an Agilent 2100 Bioanalyzer system (Agilent Technologies). All samples had RIN > 3.95 following previous established standards for RNA analysis in human brain tissue[3]. DNA contamination was assessed using primers covering an intronic region of *GAPDH*. If products were detected based on melting curve analysis, samples were DNase treated using the TURBO DNA-free Kit (Life Technologies; #AM1907) following the manufacturer’s instructions. Samples were converted to cDNA using qScript cDNA SuperMix (Quanta BioSciences; #95048) following the manufacturer’s instructions. Samples were diluted in nuclease-free H_2_O to a concentration of 150 ng/µl measured on a NanoDrop 2000C (Thermo Scientific) before aliquotation and long-term storage at -80 °C, or short-term storage at -20 °C.

## RT-qPCR

On each plate, cDNA for Human Reference Total RNA (Clontech; #636538) was included for calibration[4]. Briefly, 1 µl of samples or calibrator were added in duplicate at a concentration of 150 ng/µl to SYBR Green Master Mix (Applied Biosystems; #4309155) and primers (TAG Copenhagen), with 10 µl total volume per well. Each plate contained a standard curve made from a 7 point 1:3 dilution series of the calibrator. All primers had an efficiency of 94-101% and an R^2^>0.96. Primer concentrations were 300 or 400 nM with an annealing temperature at 56-62 °C. Samples were quantified on a QuantStudio 3 Real-Time PCR System (Thermo Fischer Scientific) with the following thermal profile: 1 cycle at 95 °C for 20 s; 45 cycles at 95 °C for 1 s followed by an annealing step with primer pair-dependent temperature for 20 s and an optional recording step with primer pair-dependent temperature for 10 s; a melting curve ranging from the primer pair-dependent annealing temperature to 95 °C. The optional recording step was included if primer dimers were observed on the melting curve in order to eliminate this signal.

## Flow Cytometry

In short, a total of 56 ml blood was collected in EDTA tubes (Greiner Bio-One; #455036) before phase separation using Ficoll-Paque PLUS (GE Healthcare Life Science; #17144003). Cells were frozen in RPMI-1640 (Sigma-Aldrich; #R5885) containing 40% FCS (Gibco; #26010-074) and 10% dimethyl sulfoxide (Sigma-Aldrich; #D4540) and stored at -130 °C. Before analysis, PBMCs were thawed, washed and centrifuged in 10% FCS RPMI-1640 for 10 min at 183 x *g* before addition of 200,000 cells per sample to a round bottom plate. Plates were spun and washed before incubation for 30 min in the dark at 4 °C with 45 µl antibody staining solution, which contained antibodies against TCR-α/β (1:8; BD Biosciences; #555548), CD3ε (1:50; BioLegend; #300431), CD4 (1:50; BD Biosciences; #564976), CD8 (1:50; BD Biosciences; #565310), CD14 (1:25; BioLegend; #325618), CD16 (1:25; BioLegend; #302012), CD45R0 (1:50; BD Biosciences; #562327), CD45RA (1:50; BD Biosciences; #563963), CD56 (1:25; BioLegend; #318334), and CD57 (1:25; BD Biosciences; #561906) in Brilliant Stain Buffer (BD Biosciences; #563794). Cells were washed and centrifuged before incubation for 15 min at room temperature with Fixation and Permeabilization Solution (Buffer A: Invitrogen; #A24217; Buffer B: Invitrogen; #A24218). Cells were resuspensed in 1% BSA (Sigma-Aldrich; #05482) in PBS for analysis on a BD LSR II flow cytometer (BD Biosciences). A minimum of 20,000 events were recorded per sample. Data were analysed using FlowJo v. 10.5.0 (BD Biosciences). Gating strategies based on Fluorescence Minus One analyses are shown in Suppl. Fig. 1, Online Resource 3. Since an age difference was apparent between groups (Table 1), data were analysed using a linear regression model that included group and age. Hoehn & Yahr[5] scores were collected for the patients.

# References

[1] L.C. Li, R. Dahiya, MethPrimer: designing primers for methylation PCRs, Bioinformatics 18(11) (2002) 1427-31.

[2] R. Rydbirk, B. Elfving, M.D. Andersen, M.A. Langbøl, J. Folke, K. Winge, B. Pakkenberg, T. Brudek, S. Aznar, Cytokine profiling in the prefrontal cortex of Parkinson's Disease and Multiple System Atrophy patients, Neurobiol Dis 106 (2017) 269-278.

[3] R. Rydbirk, J. Folke, K. Winge, S. Aznar, B. Pakkenberg, T. Brudek, Assessment of brain reference genes for RT-qPCR studies in neurodegenerative diseases, Sci Rep 6 (2016) 37116.

[4] M.W. Pfaffl, A new mathematical model for relative quantification in real-time RT–PCR, Nucleic Acids Res 29(9) (2001) e45.

[5] C.G. Goetz, W. Poewe, O. Rascol, C. Sampaio, G.T. Stebbins, C. Counsell, N. Giladi, R.G. Holloway, C.G. Moore, G.K. Wenning, M.D. Yahr, L. Seidl, Movement Disorder Society Task Force report on the Hoehn and Yahr staging scale: status and recommendations, Mov Disord 19(9) (2004) 1020-8.
