# Supplementary material for: Epigenetic modulation of AREL1 and increased HLA expression in brains of multiple system atrophy patients
Source: Acta Neuropathol Commun. 2020 Mar 9;8:29. doi: 10.1186/s40478-020-00908-7 (PMC7063795; doi:10.1186/s40478-020-00908-7)
Supplement: Supplementary file 3 — Additional file 3. Online Resource 3: Supplementary Figures. [file 40478_2020_908_MOESM3_ESM.docx]

**Epigenetic modulation of *AREL1* and increased *HLA* expression in brains of Multiple system atrophy patients**

Rasmus Rydbirk^1,2,3,a^, Jonas Folke^1,3^, Florence Busato^2^, Elodie Roché^2^, Alisha Shahzad Chauhan^1,3^, Annemette Løkkegaard^4,5^, Anne-Mette Hejl^4^, Matthias Bode^6^, Morten Blaabjerg^6,7^, Mette Møller^8^, Erik Hvid Danielsen^8^, Tomasz Brudek^1,3^, Bente Pakkenberg^1,5^, Jorg Tost^2,b^, Susana Aznar^1,3,b,^*

^1^ Research Laboratory for Stereology and Neuroscience, Bispebjerg-Frederiksberg Hospital, University Hospital of Copenhagen, Nielsine Nielsens Vej 6B, DK-2400, Copenhagen, Denmark

^2^ Laboratory for Epigenetics and Environment, Centre National de Recherche en Génomique Humaine, CEA-Institut de Biologie Francois Jacob, 2 rue Gaston Crémieux, FR-91000 Evry, France

^3^ Copenhagen Centre for Translational Research, Bispebjerg-Frederiksberg Hospital, University Hospital of Copenhagen, Nielsine Nielsens Vej 4B, Copenhagen, Denmark

^4^ Department of Neurology, Bispebjerg-Frederiksberg Hospital, University Hospital of Copenhagen, Ebba Lunds Vej 44, DK-2400, Copenhagen, Denmark.

^5^ Institute of Clinical Medicine, Faculty of Health, University of Copenhagen, Blegdamsvej 3B, DK-2200 Copenhagen, Denmark

^6^ Department of Neurology, Odense University Hospital, J.B. Winsløws Vej 4, DK-5000 Odense, Denmark.

^7^ Department of Clinical Research, University of Southern Denmark, Odense, Denmark.

^8^ Department of Neurology, Aarhus University Hospital, Aarhus, Denmark.

^a^ Present address: Biotech Research and Innovation Centre, Faculty of Health, University of Copenhagen, Denmark

^b^ Joint senior authorship

* Corresponding author:

Susana Aznar, PhD

Research Laboratory for Stereology and Neuroscience

Bispebjerg-Frederiksberg Hospital, University Hospital of Copenhagen

Nielsine Nielsens Vej 6B, stair 11B, 2nd floor

DK-2400, Copenhagen, Denmark

E-mail: [susana.aznar.kleijn@regionh.dk](mailto:susana.aznar.kleijn@regionh.dk)

# Supplementary Figures

**
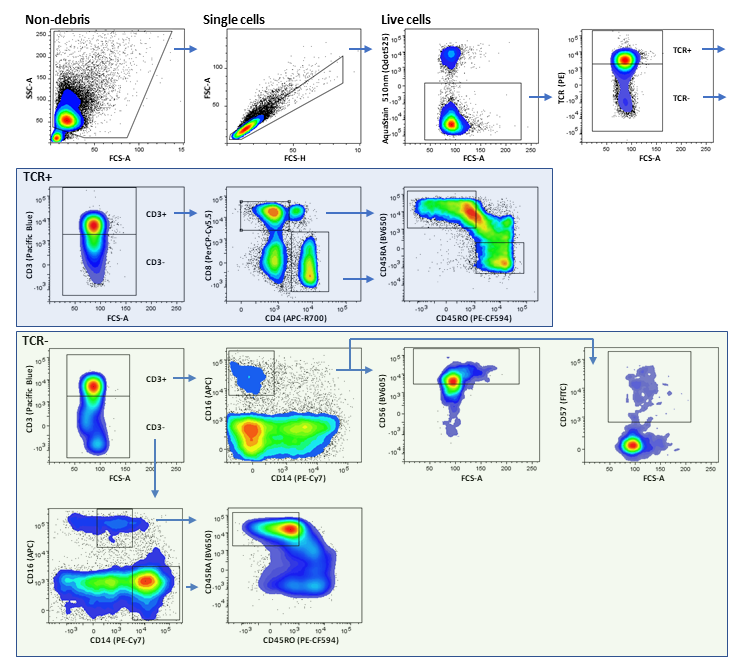
**

**Supplementary Fig. 1** Gating for flow cytometry experiment

Gating strategy employed for the flow cytometric analyses. First, gating for cell debris were used and single cells were isolated, then live cells and finally cells were divided depending on their representation of T cell receptors (TCR). For TCR^+^ cells, cells were divided depending on their presentation of CD3, then CD4 and finally CD8. For TCR^-^ cells, cells were divided according to CD3 presentation, then CD14 and CD16 followed by CD56, CD57 and/or CD45RA and/or CD45R0.


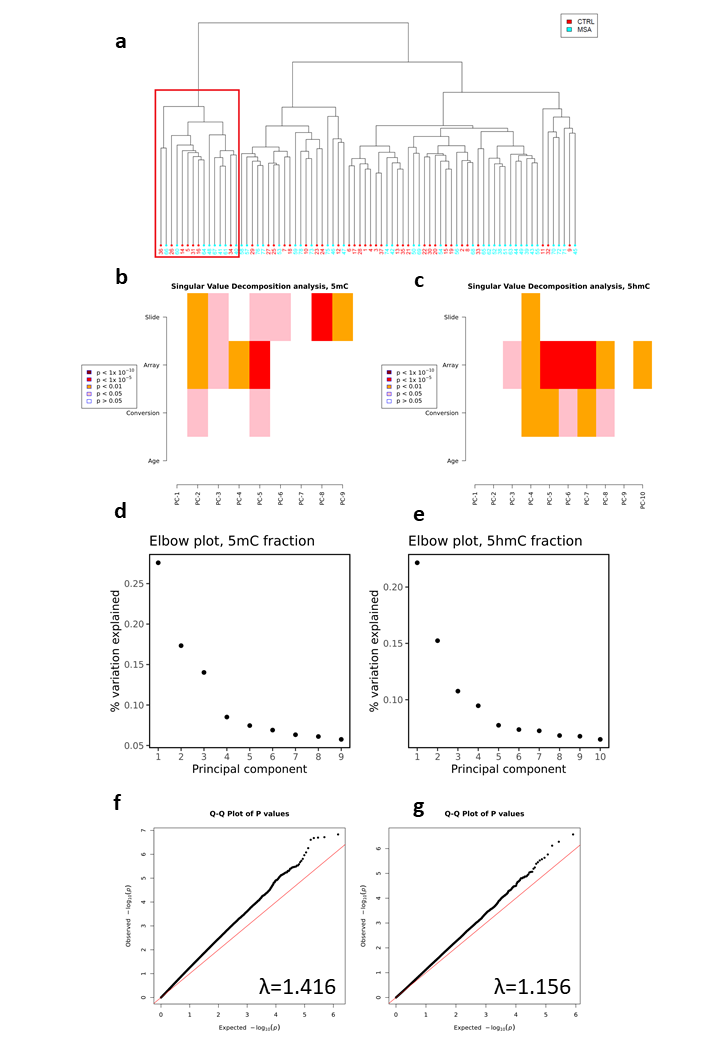


**Supplementary Fig. 2** Hierarchical clustering, SVD analyses and Q-Q plots

**a** Hierarchical clustering of samples. Outliers are marked with red square. **b-c** Single Value Decomposition analyses for putative batch effect. Array: position on MethylationEPIC BeadChip; Slide: MethylationEPIC BeadChip. Conversion: Preparation of gDNA for analysis on BeadChip. **d-e** Elbow plots showing the influence of each principal component on data. **f-g** Q-Q plots showing the P-value distribution. **b-d**-f 5-methylcytosine (5mC) fraction. **c-e-g** 5-hydroxymethylcytosine (5hmC) fraction.


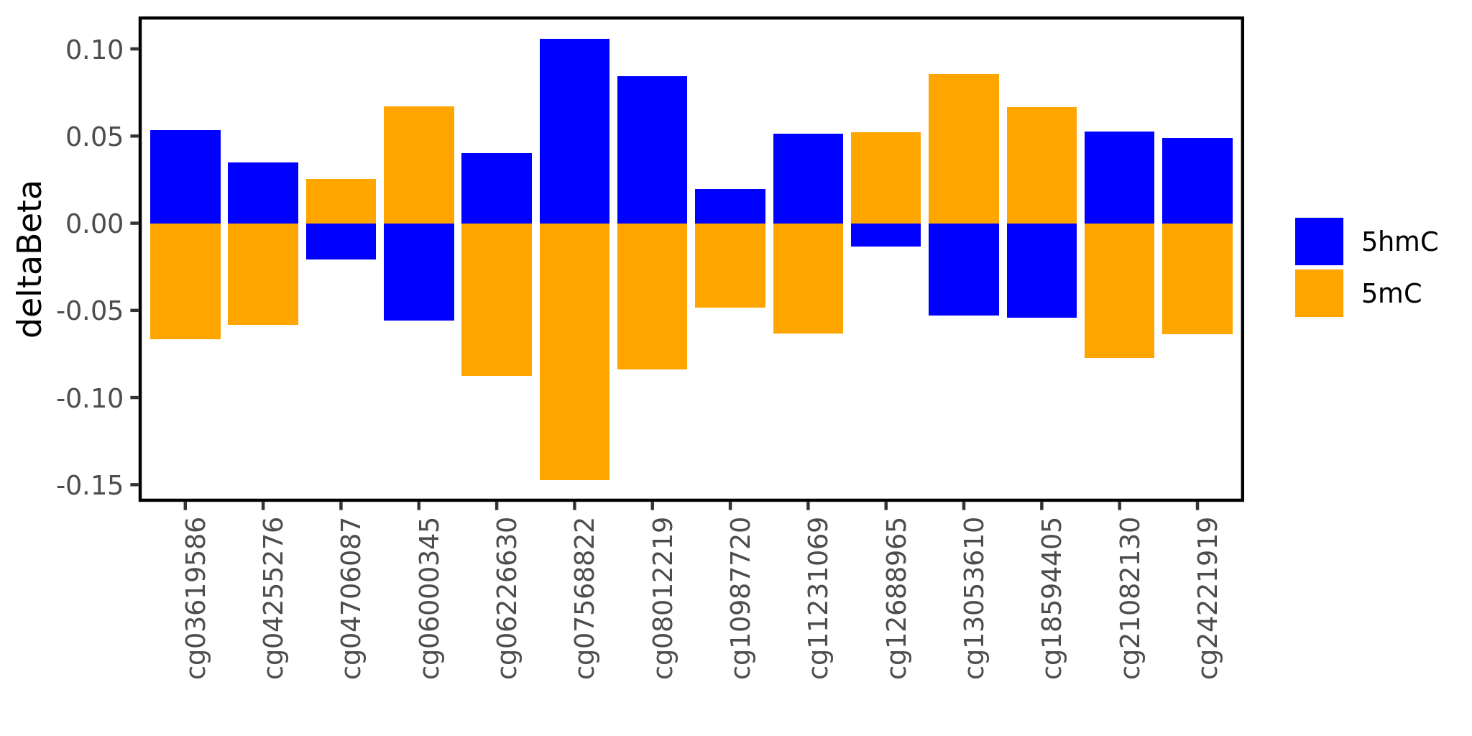


**Supplementary Fig. 3** Genes present in both 5mC and 5hmC overlapping with other EWAS studies

Probes on genes overlapping with other studies on neurodegenerative disorders. 5-methylcytosine (5mC) and 5-hydroxymethylcytosine (5hmC) changes (deltaBeta) between controls and multiple system atrophy patients are shown for each probe.


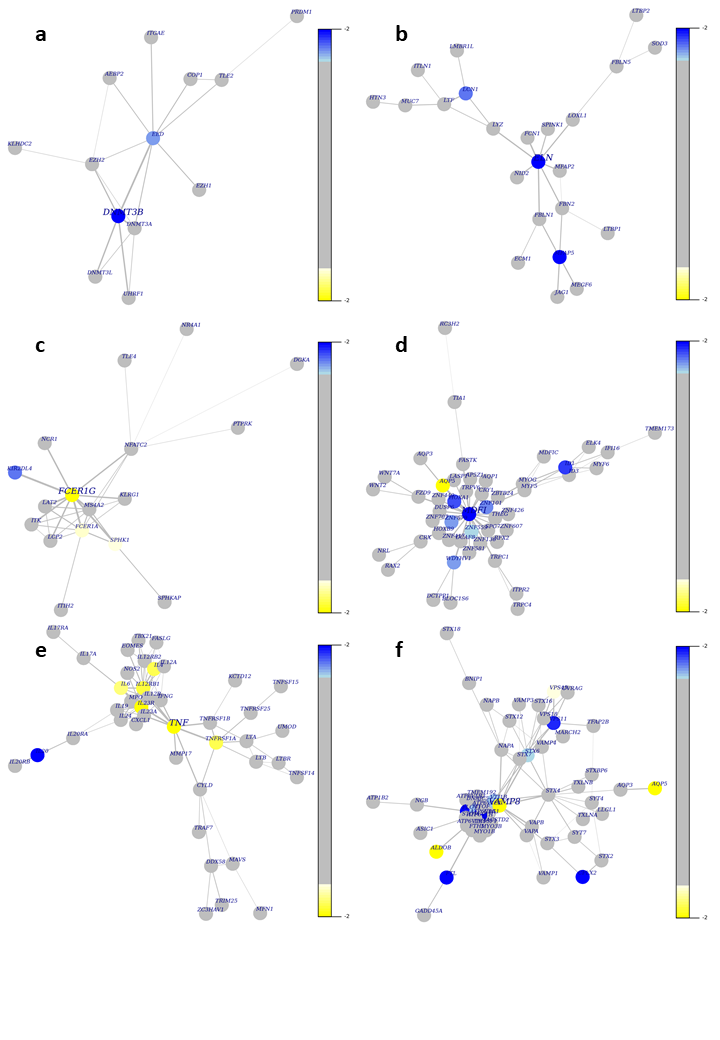


**Supplementary Fig. 4** Functional Epigenetic Modules, 5mC

Functional epigenetic modules were calculated for the 5-methylcytosine fraction. Modules for *DNMTB3* (**a**), *ELN* (**b**), *FCERG1* (**c**), *MDFI* (**d**), *TNF* (**e**), and *VAMP8* (**f**) are shown. Figures were made with the champ.EpiMod function adjusted for age and neuronal fraction. Decreased and increased methylation levels are shown with yellow and blue colours, respectively.


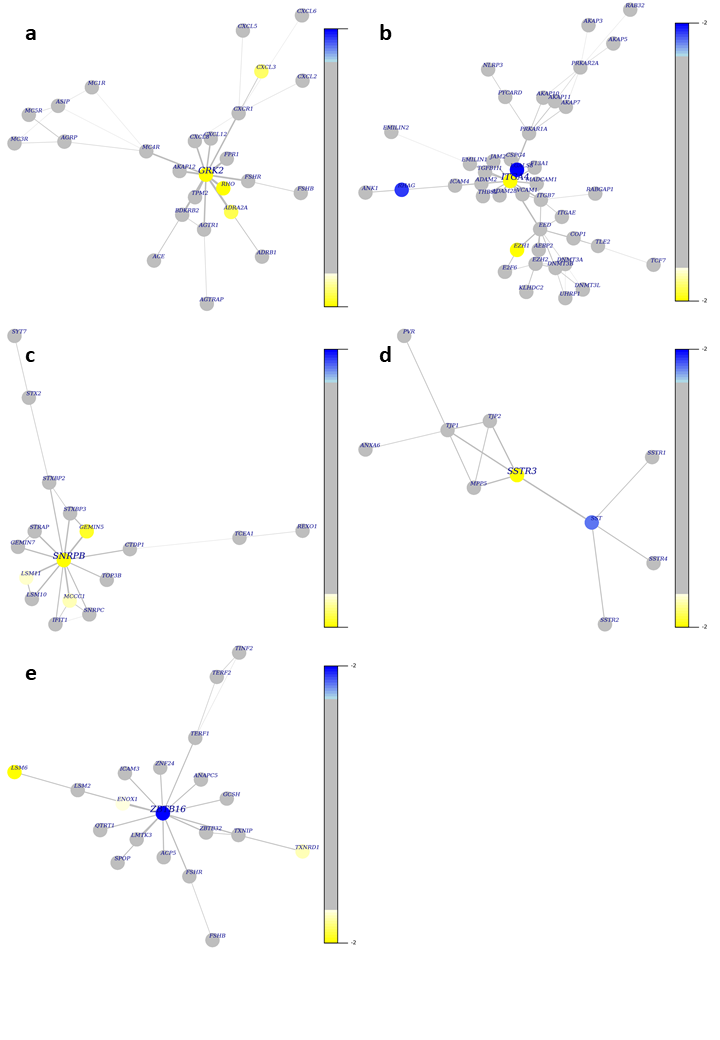


**Supplementary Fig. 5** Functional Epigenetic Modules, 5hmC

Functional epigenetic modules were calculated for the 5-hydroxymethylcytosine fraction. Modules for *GRK2* (**a**), *ITGA4* (**b**), *SNRPB* (**c**), *SSTR3* (**d**), and *ZBTB16* (**e**) are shown. Figures were made with champ.EpiMod function adjusted for age and neuronal fraction. Decreased and increased methylation levels are shown with yellow and blue colours, respectively.
